# An interpretable nomogram for predicting early acute postoperative hypocalcemia in differentiated thyroid cancer: development and internal validation

**DOI:** 10.3389/fendo.2026.1840443

**Published:** 2026-06-17

**Authors:** Fangfang Zhong, Changqing Zhou, Lijiao Zhang, Wenwen Chen, Qianqian Yang, Danying Liu, Yun Zhu, Lei Xie

**Affiliations:** 1 Nursing Department, Sir Run Run Shaw Hospital, Zhejiang University School of Medicine, Hangzhou, China; 2 Department of Head and Neck Surgery, Sir Run Run Shaw Hospital, Zhejiang University School of Medicine, Hangzhou, China

**Keywords:** autologous parathyroid transplantation, clinical prediction model, differentiated thyroid cancer, machine learning, nomogram, postoperative hypocalcemia, SHAP, thyroidectomy

## Abstract

**Background:**

Postoperative hypocalcemia (HC) is a frequent complication following thyroidectomy. Balancing prophylactic calcium supplementation against avoiding overtreatment remains a clinical dilemma. This study aimed to develop and independently validate an interpretable machine learning (ML) model to predict early acute postoperative HC in patients with differentiated thyroid cancer (DTC).

**Methods:**

In accordance with the Transparent Reporting of a multivariable prediction model for Individual Prognosis or Diagnosis plus Artificial Intelligence reporting guideline, diverse clinical, biochemical, and surgical parameters from 876 DTC patients (February 2023–January 2026) were retrospectively analyzed. Early acute postoperative HC was defined as biochemical or symptomatic hypocalcemia occurring within the first 24 hours after surgery. The cohort was randomly split into a training set (80%, n = 701) and an independent holdout test set (20%, n = 175). Seven ML algorithms were trained and evaluated. Shapley Additive exPlanations (SHAP) and a clinical nomogram were utilized for model interpretation and clinical application.

**Results:**

The overall incidence of early acute postoperative HC was 38.2% (335/876). While complex ensemble models showed comparable performance, Logistic Regression (LR) was selected as the final model for its optimal balance of favorable discrimination (area under the receiver operating characteristic curve in the test set = 0.760) and clinical transparency. Multivariate and SHAP analyses revealed that preoperative serum magnesium, body mass index, lateral cervical lymph node dissection, and explicit parathyroid autotransplantation dynamics (specifically implantation into the neck muscle, odds ratio = 6.808) were critical drivers of HC. The LR-derived nomogram demonstrated excellent sensitivity (0.806 in the test set) at the optimal threshold of 0.372. Decision curve analysis confirmed superior net clinical benefit across relevant threshold probabilities, and subgroup analyses verified the model’s stability across age and sex strata.

**Conclusions:**

We developed and independently validated a transparent, highly discriminative LR-based nomogram for predicting early acute postoperative HC within the first 24 hours after thyroidectomy. Integrating precise surgical factors and biochemical profiles, this tool facilitates individualized perioperative monitoring and tailored calcium supplementation strategies.

## 1 Introduction

Thyroid cancer is the most common malignancy of the endocrine system. In 2022, the number of new thyroid cancer cases in China reached 466,100, ranking it the third most frequent cancer in terms of incidence (1). Currently, surgical resection—ranging from hemithyroidectomy to total thyroidectomy, often accompanied by central or lateral neck dissection—remains the primary therapeutic mainstay for differentiated thyroid cancer. However, despite advancements in refined surgical techniques and parathyroid protection strategies, postoperative hypocalcemia (HC) is still one of the most common complications following thyroid cancer surgery, with reported rates varying between 25% and 39.6% (2, 3). HC encompasses a broad clinical spectrum, ranging from subtle sensory disturbances to life-threatening emergencies. Initial manifestations typically present as perioral and acral paresthesia. Mechanistically, HC lowers the depolarization threshold of neurons and myocytes, thereby exacerbating neuromuscular irritability and predisposing to cardiac electrophysiological instability. While mild cases are characterized by muscle stiffness and cramps, progressive HC may precipitate tetany, laryngospasm, and neuropsychiatric sequelae, including cognitive impairment, psychological disorders and seizures. Furthermore, chronic or recurrent HC disrupts mineral homeostasis, potentially inducing multi-organ dysfunction. Significantly, QTc interval prolongation increases the vulnerability to torsades de pointes and fatal ventricular fibrillation (4). Consequently, HC significantly exacerbates patient distress and complicates therapeutic management. The heightened demand for supplemental medical and nursing care inevitably prolongs the length of stay (LOS) and escalates healthcare expenditures. Beyond acute outcomes and diminished quality of life (QoL), HC also predisposes patients to long-term sequelae, such as chronic renal insufficiency and basal ganglia calcification (5).

However, unwarranted and excessive calcium supplementation can lead to complications such as nephrolithiasis and heightened cardiovascular risks, including myocardial infarction, and systemic ectopic calcification (6). Consequently, managing postoperative hypocalcemia remains one of the most challenging therapeutic paradoxes in clinical care. Hence, early risk identification and personalized supplementation are vital for ensuring safe discharge, optimizing bed turnover, and enhancing cost-effectiveness.

Although numerous studies have investigated risk factors for postoperative HC following thyroid cancer surgery, findings have been inconsistent. Reported determinants encompass surgical variables (e.g., parathyroid gland injury (7), total thyroidectomy (8), extent of lymph node dissection (9), and the use of intraoperative tracer techniques (10)), patient-related factors (e.g., perioperative parathyroid hormone [PTH] levels (11), sex (9), body mass index [BMI] (12), age), and tumor characteristics (2). Nevertheless, no single predictor has demonstrated sufficient discriminative ability for reliable individualized risk stratification.

Clinical prediction models (CPMs) are essential risk stratification tools that estimate individual disease probabilities by integrating clinicopathological variables. Recently, machine learning (ML) has advanced medical modeling by efficiently processing high-dimensional data and capturing non-linear relationships to enhance predictive accuracy (13). Recent studies have also demonstrated the feasibility of interpretable machine learning approaches in clinical risk prediction, including cancer survival and mortality modeling (14, 15). However, ML’s inherent complexity often creates uninterpretable ‘black-box’ models. Shapley Additive Explanations (SHAP) resolves this by providing both local and global interpretations of feature contributions, thereby restoring model transparency (16).

Currently, predictive models for postoperative HC in thyroid cancer remain limited and lack consensus regarding predictor selection. Most models rely on isolated laboratory parameters rather than integrating comprehensive demographic, surgical, and pathological variables. Furthermore, they are frequently constrained by limited sample sizes and the lack of independent external or holdout testing setups (17, 18)—a crucial requirement to mitigate the biases inherent in retrospective, non-randomized designs (19). More importantly, while inadvertent parathyroid gland resection or devascularization is widely recognized as the primary mechanism of HC, the highly nuanced intraoperative details—specifically the quantity and the exact anatomical site of parathyroid autotransplantation—have been largely overlooked in existing predictive frameworks. Additionally, existing literature frequently defines the outcome based solely on laboratory parameters (biochemical HC), overlooking patients who develop clinical symptoms despite normal serum calcium levels (symptomatic HC) (20). To ensure scientific rigor and clinical relevance, this study adopts a comprehensive definition of HC that encompasses both biochemical criteria (albumin-corrected calcium < 2.0 mmol/L (21)) and symptomatic manifestations (22). This inclusive approach reduces outcome misclassification and aligns closely with real-world requirements for early clinical intervention. Despite their widespread success as intuitive risk stratification tools in oncology, nomograms remain underutilized for HC prediction.

Therefore, this study aims to develop an ML-based prediction model for early acute postoperative HC within the first 24 hours after thyroidectomy by integrating comprehensive clinical variables. By adopting an inclusive definition of HC, identifying the optimal ML algorithm, employing SHAP for model transparency, and constructing a clinical nomogram, this study provides a robust, clinically relevant tool for clinicians for early risk identification and tailored preventive strategies.

## 2 Methods

### 2.1 Study design and ethical statement

This retrospective cohort study was conducted at Sir Run Run Shaw Hospital, Zhejiang University School of Medicine (a tertiary care, academic referral, and university-affiliated hospital) between February, 2023 and January, 2026.The study protocol was approved by the Medical Ethics Committee of Sir Run Run Shaw Hospital, Zhejiang University School of Medicine (Approval No. [20220228]) and was performed in accordance with the Declaration of Helsinki. Written informed consent was waived due to the retrospective nature of the study. Finally, this study adheres to the newly proposed TRIPOD+AI guideline (13) for the reporting of machine learning-based prediction models.

### 2.2 Sample size estimation and statistical power

To prevent overfitting and ensure stable model derivation, we evaluated sample size adequacy according to established methodological criteria. For machine learning-based multivariable prediction models, an events-per-variable (EPV) ratio of at least 10 is widely recommended. In our study, the 15 selected clinical variables were transformed (e.g., via one-hot encoding for multi-category surgical factors) into 21 independent predictor parameters, dictating a requirement of at least 210 target events. Based on published literature, the expected incidence of postoperative hypocalcemia ranges from 25.0% to 39.6%. Even aiming for 210 events under the most conservative incidence assumption (25.0%) would necessitate a minimum cohort of 840 patients. Our final curated cohort encompassed 876 patients with 335 actual events (observed incidence: 38.2%), yielding an actual EPV of 16.0, substantially exceeding the conventional threshold. Furthermore, this large absolute number of outcome events (n = 335) ensures sufficient statistical power to achieve precise estimation of overall risk and stable coefficient derivation, thereby mitigating the risk of high-dimensional overfitting. This sample size and event count satisfy the minimal requirements for developing and validating multivariable prediction models, supporting the reliability and generalizability of the final model.

### 2.3 Study population

Inclusion criteria were defined as follows:(1) Patients aged ≥ 18 years who underwent initial thyroidectomy (unilateral or bilateral) at our institution;(2) Postoperative pathology confirming differentiated thyroid cancer (DTC, including papillary or follicular thyroid carcinoma);(3) Intraoperative rapid frozen section pathology performed to confirm the diagnosis;(4) Comprehensive baseline evaluations upon admission, including preoperative serum calcium and parathyroid function tests;(5) No history of long-term use of medications affecting serum calcium or parathyroid hormone (PTH) metabolism prior to surgery; and(6) Availability of complete medical, operative records (strictly requiring complete records of both intraoperative details).

Exclusion criteria included: (1) Pathological diagnosis of medullary, anaplastic, or other rare types of undifferentiated thyroid carcinoma (to eliminate pathophysiological confounding from intrinsic calcitonin secretion and avoid inherent selection bias associated with their routinely more extensive surgical management); (2) A history of non-thyroidal neck disease or neck surgery;(3) Preoperative coexistence of other diseases affecting serum calcium levels and/or parathyroid function; and (4) Severe hepatic or renal insufficiency, or the presence of bone metabolic disorders (e.g., severe osteoporosis or vitamin D deficiency).

**Figure 1:**
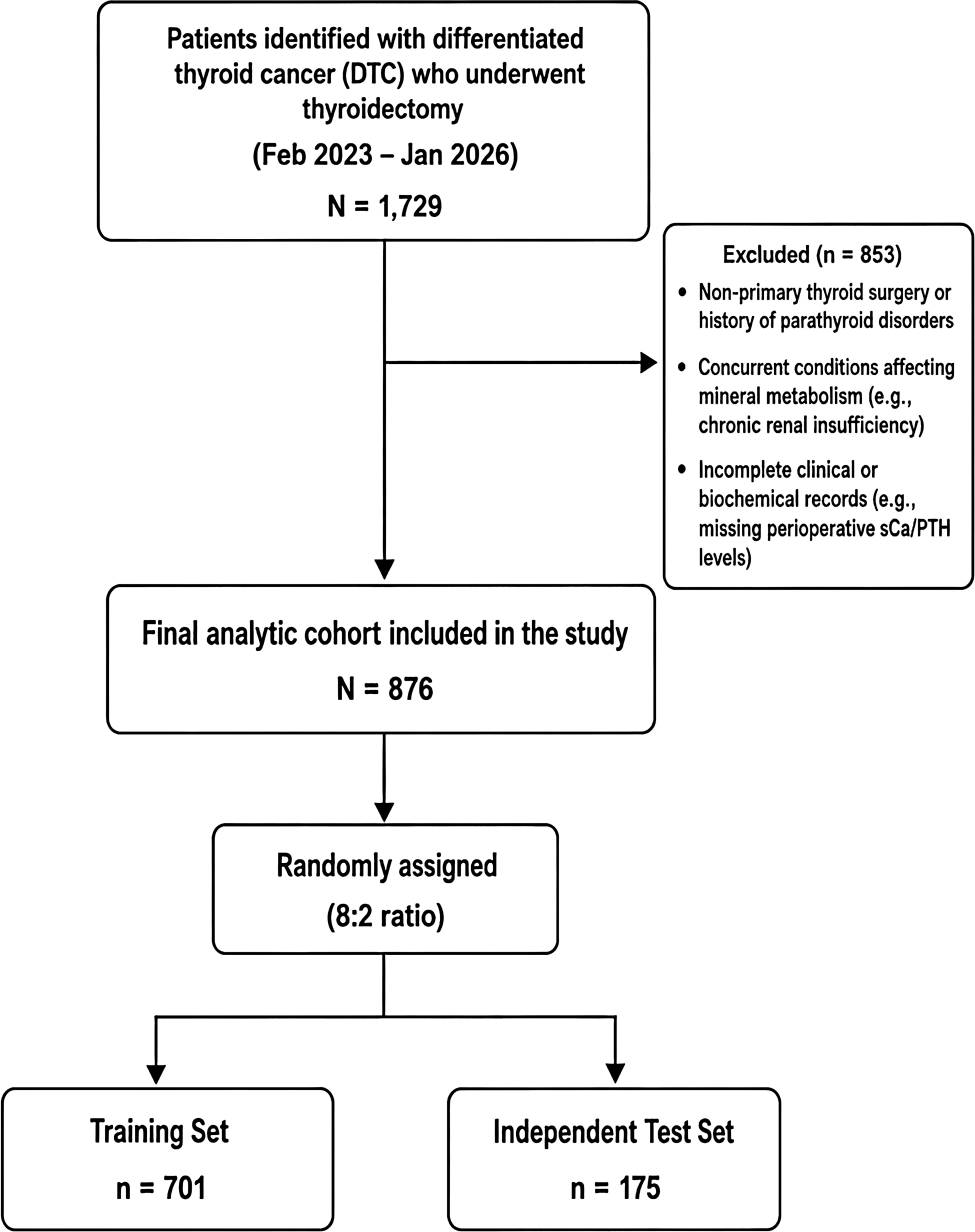
Flowchart of participant selection. DTC, differentiated thyroid cancer; sCa, serum calcium; PTH, parathyroid hormone.

Current study population and the selection workflow are summarized in **Figure 1**.

### 2.4 Surgical procedures and perioperative management

All surgical procedures were performed consistently by the senior surgical team experienced in thyroidectomy. According to clinical guidelines, procedures included unilateral or bilateral thyroidectomy, accompanied by prophylactic or therapeutic central compartment lymph node dissection (CCLND). Lateral cervical lymph node dissection (LCLND) was performed only when biopsy-proven metastatic lymphadenopathy was confirmed. Intraoperative tracers (e.g., carbon nanoparticles, indocyanine green, Methylene Blue or Mitoxantrone) were utilized in a subset of patients to facilitate the identification and preservation of parathyroid glands (PGs), whereas intraoperative PTH monitoring was not employed. Any PG tissue showing signs of devascularization or compromised blood supply was autotransplanted. For all patients, serum calcium and PTH concentrations were routinely measured on the first postoperative morning.

### 2.5 Data collection and candidate predictors

Clinical data were systematically extracted from the electronic medical record system, including both medical and nursing records. Candidate variables were identified through a comprehensive literature review and multidisciplinary consultations with a panel of seven specialists in clinical medicine, nursing, and statistics. Based on expert consensus and clinical relevance, the candidate variables were systematically classified into five domains:

(1) Demographic and clinical features: Age, sex, body mass index (BMI), smoking and alcohol consumption status, allergy history, and comorbidities (e.g., hypertension, diabetes mellitus). (2) Surgical factors: Surgery laterality (categorized as left-sided, right-sided, bilateral), execution of lateral cervical lymph node dissection (yes/no), and parathyroid autotransplantation details (number of grafted parathyroid glands; graft site: non-grafted, cervical muscle, presternal/adipose tissue, forearm muscle, or combined multi-site). (3) Pathological characteristics: Tumor focality (solitary vs. multiple) and count of cancer foci. (4) Perioperative management: Prophylactic calcium supplementation (yes/no). (5) Baseline biochemical indicators: Preoperative levels of serum calcium, magnesium, and parathyroid hormone (PTH).

The endpoint was early acute postoperative hypocalcemia, defined as hypocalcemia occurring within the first 24 hours after surgery and framed as a binary outcome (yes/no). To ensure temporal consistency, baseline biochemical values were strictly determined based on the last laboratory measurement recorded prior to surgery. The postoperative endpoint was determined using laboratory results obtained within the first 24 hours following the operation. In cases where multiple postoperative tests were available within this specific time window, the mean value was used for outcome definition. Accordingly, this model should be interpreted as predicting early acute postoperative HC rather than delayed, persistent, or chronic hypocalcemia.

### 2.6 Assessment of hypocalcemia

Early acute postoperative HC was strictly defined within the first 24 hours after surgery as either: (1) a minimum albumin-corrected serum total calcium level of strictly < 2.0 mmol/L after surgery (biochemical HC), or (2) the manifestation of typical hypocalcemia symptoms (e.g., perioral numbness, tingling in the extremities, or positive Chvostek/Trousseau signs) necessitating clinical intervention, regardless of the absolute serum calcium concentration (symptomatic HC).

### 2.7 Quality control

Rigorous quality control measures were implemented during data extraction. All data were double-entered independently by two researchers. Discrepancies were resolved by consensus or by consulting a third senior investigator, who reviewed the original records to ensure data accuracy and consistency. Postoperative pathology was confirmed by at least two independent expert pathologists.

## 3 Statistical analysis

### 3.1 Descriptive statistics and data preprocessing

Continuous variables were assessed for normality using the Kolmogorov-Smirnov test. Normally distributed continuous variables were presented as mean ± standard deviation (SD) and compared using the independent-samples t-test. Non-normally distributed continuous data were expressed as median with interquartile range (IQR) and compared using the Mann-Whitney U test. Categorical variables were presented as frequencies and percentages (%), and between-group differences were evaluated using the Pearson χ² test or Fisher’s exact test, as appropriate.

To ensure an unbiased evaluation of the model’s generalizability, the complete dataset (N = 876) was randomly partitioned into a training cohort (80%, n = 701) and an independent internal holdout test cohort (20%, n = 175) using stratified sampling to preserve the outcome prevalence. The test set was strictly sequestered and did not participate in any model training, feature selection, or hyperparameter tuning processes. To strictly prevent data leakage, all preprocessing steps were encapsulated within the cross-validation loops, meaning transformation parameters were estimated exclusively on the training folds and subsequently applied to the validation or hold-out test sets. Missing continuous variables were imputed using the median, while categorical variables were imputed via the mode. Continuous features (e.g., age, body mass index, and preoperative biochemical indices) were standardized. Multi-category surgical descriptors, such as surgery laterality and parathyroid autotransplantation site, underwent one-hot encoding without imposing artificial ordinality. All dichotomous variables were unified into a 0/1 scheme. Age and sex were maintained as stratification variables for downstream bias and subgroup evaluation.

### 3.2 Machine learning pipeline and model development

Seven machine learning algorithms were evaluated: Logistic Regression (LR) with regularization, Support Vector Machine (SVM), Random Forest (RF), eXtreme Gradient Boosting (XGBoost), Light Gradient Boosting Machine (LightGBM), Categorical Boosting (CatBoost), and an Artificial Neural Network (ANN). Model training and hyperparameter tuning were rigorously restricted to the training set via internal stratified k-fold cross-validation with grid search to optimize hyperparameters for each algorithm, optimizing for the area under the receiver operating characteristic curve (AUC). Model out-of-fold (OOF) predictions from the cross-validation were used to assess internal performance, thereby minimizing in-sample optimism bias.

### 3.3 Model performance evaluation and validation

Final generalizability was evaluated on the sequestered test set. Discrimination was evaluated using the area under the receiver operating characteristic (ROC) curves and the precision-recall (PR) curves. Operating thresholds were determined using the Youden index derived from the training OOF ROC curve and rigidly applied to the test set to calculate sensitivity, specificity, accuracy, positive predictive value (PPV), negative predictive value (NPV). Clinical utility was assessed using Decision Curve Analysis (DCA) to estimate the net benefit across a range of threshold probabilities. Furthermore, we conducted subgroup analyses stratified by sex and age tertiles to evaluate potential performance disparities. For model interpretability, Shapley Additive exPlanations (SHAP) with a specific focus on global feature importance was employed to elucidate the mechanism underlying the predictions.

### 3.4 Statistical software and environment

Baseline descriptive statistical analyses were conducted using SPSS software (version 26.0, IBM Corp., Armonk, NY, USA). All algorithm derivation and machine learning evaluations were performed utilizing the Python programming language (version 3.10.18, Python Software Foundation). Specifically, conventional models and data resampling pipelines were implemented via the scikit-learn package (version 1.7.2). Advanced gradient boosting frameworks were executed utilizing the xgboost, lightgbm, and catboost libraries. Model interpretability algorithms were conducted via the shap package. Custom scripts leveraging numpy and pandas were utilized for metric computations and DCA. All statistical tests were two-sided, and a P-value < 0.05 was considered statistically significant.

## 4 Results

### 4.1 Baseline characteristics and incidence of early acute postoperative hypocalcemia

In total, 876 patients were included in this study. Early acute postoperative hypocalcemia occurred in 335 patients, yielding an overall incidence of 38.2%. The baseline clinical, pathological, and surgical characteristics of the cohort are summarized in **Table 1**. Compared with the non-hypocalcemia group (n = 541), patients in the postoperative hypocalcemia group (n = 335) were significantly more likely to be female (*p* = 0.002), have a lower body mass index (BMI) (*p* < 0.001), present with fewer comorbidities (*p* = 0.016), and have a lower rate of smoking and alcohol consumption (*p* = 0.041).

**Table 1:** Comparison of clinical and operative characteristics between patients with and without early acute postoperative hypocalcemia.

Variable	HC group (n = 335)	Non-HC group (n = 541)	Statistic	*p*
Age (years) [M (Q1-Q3)]	44.00 (33.00-55.00)	46.00 (33.00-56.00)	96019.500	0.138
Sex			9.906	0.002
Male	64 (19.1%)	156 (28.8%)		
Female	271 (80.9%)	385 (71.2%)		
Body mass index (kg/m²) [M (Q1-Q3)]	23.10 (20.80-25.40)	24.20 (21.60-26.60)	102946.000	**< 0.001**
History of smoking and alcohol use			4.170	0.041
Yes	25 (7.5%)	65 (12.0%)		
No	310 (92.5%)	476 (88.0%)		
History of allergy			0.128	0.721
Yes	44 (13.1%)	77 (14.2%)		
No	291 (86.9%)	464 (85.8%)		
Comorbidities			5.852	0.016
Yes	134 (40.0%)	263 (48.6%)		
No	201 (60.0%)	278 (51.4%)		
Preoperative serum calcium (mmol/L) [M (Q1-Q3)]	2.38 (2.32-2.43)	2.40 (2.34-2.45)	104159.000	**< 0.001**
Preoperative serum magnesium (mmol/L) [M (Q1-Q3)]	0.87 (0.83-0.91)	0.88 (0.84-0.92)	98289.000	**0.035**
Preoperative parathyroid hormone (pg/mL) [M (Q1-Q3)]	41.70 (32.95-51.10)	40.70 (32.10-50.80)	88569.000	0.574
Side of surgery [n (%)] ^a^			149.585	< 0.001
Left	7 (2.1%)	129 (24.0%)		
Right	17 (5.1%)	121 (22.5%)		
Bilateral	311 (92.8%)	288 (53.5%)		
Lateral cervical lymph node dissection			90.809	< 0.001
Yes	163 (48.7%)	98 (18.1%)		
No	172 (51.3%)	443 (81.9%)		
Number of tumor foci [M (Q1-Q3)]	2.00 (1.00-3.00)	1.00 (1.00-2.00)	71951.500	**< 0.001**
Number of autotransplanted parathyroid glands [M (Q1-Q3)]	1.00 (1.00-2.00)	1.00 (1.00-1.00)	63824.500	**< 0.001**
Site of parathyroid autotransplantation			101.592	< 0.001
Non-transplanted	41 (12.2%)	38 (7.0%)		
Neck muscle	13 (3.9%)	7 (1.3%)		
Combined/Multiple sites	149 (44.5%)	407 (75.2%)		
Presternal/Fat	67 (20.0%)	22 (4.1%)		
Forearm muscle	65 (19.4%)	67 (12.4%)		
Prophylactic calcium supplementation			37.031	< 0.001
Yes	251 (74.9%)	293 (54.2%)		
No	84 (25.1%)	248 (45.8%)		

Data are presented as median (IQR) or n (%). ^a^ Excluding 3 patients with isthmectomy. Bold *p* values denote statistical significance (*p* < 0.05). HC, hypocalcemia; IQR, interquartile range; PTH, parathyroid hormone.

Regarding surgical and pathological parameters, the hypocalcemia group exhibited a significantly higher structural disease burden and more extensive surgical interventions. Specifically, patients developing hypocalcemia had a higher median number of cancer foci (*p* < 0.001), a significantly higher proportion of bilateral thyroidectomy (92.8% vs. 53.5%, *p* < 0.001), and higher rates of lateral cervical lymph node dissection (LCLND) (48.7% vs. 18.1%, *p* < 0.001). Furthermore, the number of parathyroid autotransplantations and the specific transplantation sites showed significant differences between the two groups (both *p* < 0.001). Baseline preoperative serum calcium (*p* < 0.001) and magnesium (*p* = 0.035) levels were slightly but significantly lower in the hypocalcemia group. Conversely, no significant differences were observed in age, allergy history, or preoperative parathyroid hormone (PTH) levels (all *p* > 0.05).

### 4.2 Univariate and multivariate analyses for early acute postoperative hypocalcemia

In the univariate analysis (**Table 2**), male sex, higher body mass index (BMI), and certain baseline characteristics (such as comorbidities and a positive history of smoking and alcohol use) were associated with a reduced incidence of HC. Conversely, aggressive disease features and surgical interventions significantly elevated the risk. Specifically, LCLND (OR = 4.284, *p* < 0.001), increased tumor foci, a higher number of autotransplanted parathyroid glands, and prophylactic calcium supplementation were strongly associated with HC occurrence (all *p* < 0.001). Regarding surgical extent, consistent with clinical expectations, bilateral thyroidectomy posed a substantially higher risk compared to unilateral procedures. For parathyroid autotransplantation (PAT), implantation in the presternal region/fat tissue increased the risk compared to non-transplanted patients (OR = 2.823, *p* = 0.002), while a combined-site approach showed a protective effect.

**Table 2:** Univariate logistic regression analysis of factors associated with early acute postoperative hypocalcemia.

Variable	OR	95% CI	Wald χ²	*p* value
Age (years)	0.993	0.983-1.003	1.828	0.176
Sex (Male vs. Female)	0.583	0.419-0.811	10.290	**0.001**
Body mass index (kg/m²)	0.939	0.905-0.975	10.581	**0.001**
History of smoking and alcohol use (Yes vs. No)	0.591	0.364-0.957	4.569	0.033
History of allergy (Yes vs. No)	0.911	0.612-1.357	0.210	0.647
Comorbidities (Yes vs. No)	0.705	0.535-0.929	6.175	**0.013**
Preoperative serum calcium (mmol/L)	1.062	0.917-1.230	0.643	0.423
Preoperative serum magnesium (mmol/L)	0.298	0.036-2.465	1.260	0.262
Preoperative parathyroid hormone (pg/mL)	1.001	0.993-1.009	0.083	0.773
Surgical laterality ^a^			174.910	< 0.001
Bilateral	1.00 (Reference)	—	—	**—**
Left	0.050	0.023-0.109	56.864	**< 0.001**
Right	0.130	0.076-0.222	56.376	**< 0.001**
Lateral neck lymph node dissection (Yes vs. No)	4.284	3.154-5.819	86.710	**< 0.001**
Number of tumor foci	1.263	1.150-1.386	—	**< 0.001**
Number of autotransplanted parathyroid glands	2.739	2.145-3.498	65.283	**< 0.001**
Site of parathyroid autotransplantation			101.346	< 0.001
Non-transplanted	1.00 (Reference)	—	—	—
Combined/multiple sites	0.339	0.210-0.548	19.512	**< 0.001**
Neck muscle	1.721	0.621-4.770	1.090	0.296
Presternal region/fat tissue	2.823	1.469-5.424	9.693	**0.002**
Forearm muscle	0.899	0.515-1.571	0.139	0.709
Prophylactic calcium supplementation (Yes vs. No)	2.529	1.875-3.412	36.898	**< 0.001**

OR, odds ratio; CI, confidence interval. ^a^Excluding 3 patients with isthmectomy. Bold *p* values denote statistical significance (*p* < 0.05).

To adjust for potential confounders, variables demonstrating statistical significance (*p* < 0.05) in the univariate model were included in the multivariate logistic regression analysis (**Table 3**). After systematic adjustment, LCLND was confirmed as a robust independent risk factor for HC (OR = 2.185, 95% CI: 1.466–3.256, *p* < 0.001). Male sex, higher BMI, and unilateral surgery consistently maintained their independent protective effects against HC.

**Table 3:** Multivariate logistic regression analysis of factors associated with early acute postoperative hypocalcemia.

Variable	β	SE	OR	95% CI	Wald χ²	*p* value
Constant	0.923	0.716	—	—	1.663	0.197
Sex (Male vs. Female)	-0.761	0.208	0.467	0.311-0.703	13.347	**< 0.001**
Body mass index (kg/m²)	-0.064	0.023	0.938	0.896-0.981	7.705	**0.006**
Lateral neck lymph node dissection (Yes vs. No)	0.782	0.203	2.185	1.466-3.256	14.754	**< 0.001**
Number of tumor foci	0.025	0.052	1.026	0.926-1.137	0.235	0.628
Number of autotransplanted parathyroid glands	0.344	0.184	1.410	0.983-2.024	3.482	0.062
Prophylactic calcium supplementation (Yes vs. No)	0.248	0.198	1.282	0.869-1.891	1.568	0.211
Surgical laterality ^a^						
Bilateral (Reference)	—	—	1.00	—	—	—
Left	-2.291	0.416	0.101	0.045-0.229	30.369	**< 0.001**
Right	-1.554	0.298	0.211	0.118-0.379	27.258	**< 0.001**
Site of parathyroid autotransplantation						
Non-transplanted (Reference)	—	—	1.00	—	—	—
Neck muscle	1.918	0.753	6.808	1.557-29.759	6.496	**0.011**
Combined/multiple sites	-0.388	0.307	0.678	0.372-1.237	1.602	0.206
Presternal region/fat tissue	0.478	0.372	1.613	0.779-3.342	1.656	0.198
Forearm muscle	-0.279	0.352	0.756	0.379-1.509	0.629	0.428

β, regression coefficient; CI, confidence interval; OR, odds ratio; SE, standard error. Covariates included in the model were sex, body mass index, lateral neck lymph node dissection, number of tumor foci, number of autotransplanted parathyroid glands, prophylactic calcium supplementation, surgical laterality, and site of parathyroid autotransplantation. ^a^Excluding 3 patients with isthmectomy. Bold *p* values denote statistical significance (*p* < 0.05).

Most notably, the statistical impact of PAT sites shifted significantly in the multivariate model. Compared with the non-transplanted patients, PAT specifically into the neck muscle emerged as a highly significant independent risk factor for HC (OR = 6.808, 95% CI: 1.557–29.759, *p* = 0.011). However, baseline comorbidities, smoking/alcohol history, tumor foci count, number of autotransplanted glands, prophylactic calcium use, and all other specific PAT sites lost their statistical significance in the final model (all *p* > 0.05).

### 4.3 Development and validation of machine learning predictive models

To approximate real-world deployment, the complete dataset of 876 patients was randomly partitioned into a training set (80%, n = 701) and an independent holdout test set (20%, n = 175) using stratified sampling to preserve the prevalence of early acute postoperative HC. Seven learning algorithms—Logistic Regression (LR), Support Vector Machine (SVM), Random Forest (RF), XGBoost, LightGBM, CatBoost, and Artificial Neural Network (ANN)—were trained and evaluated.

**Figure 2:**
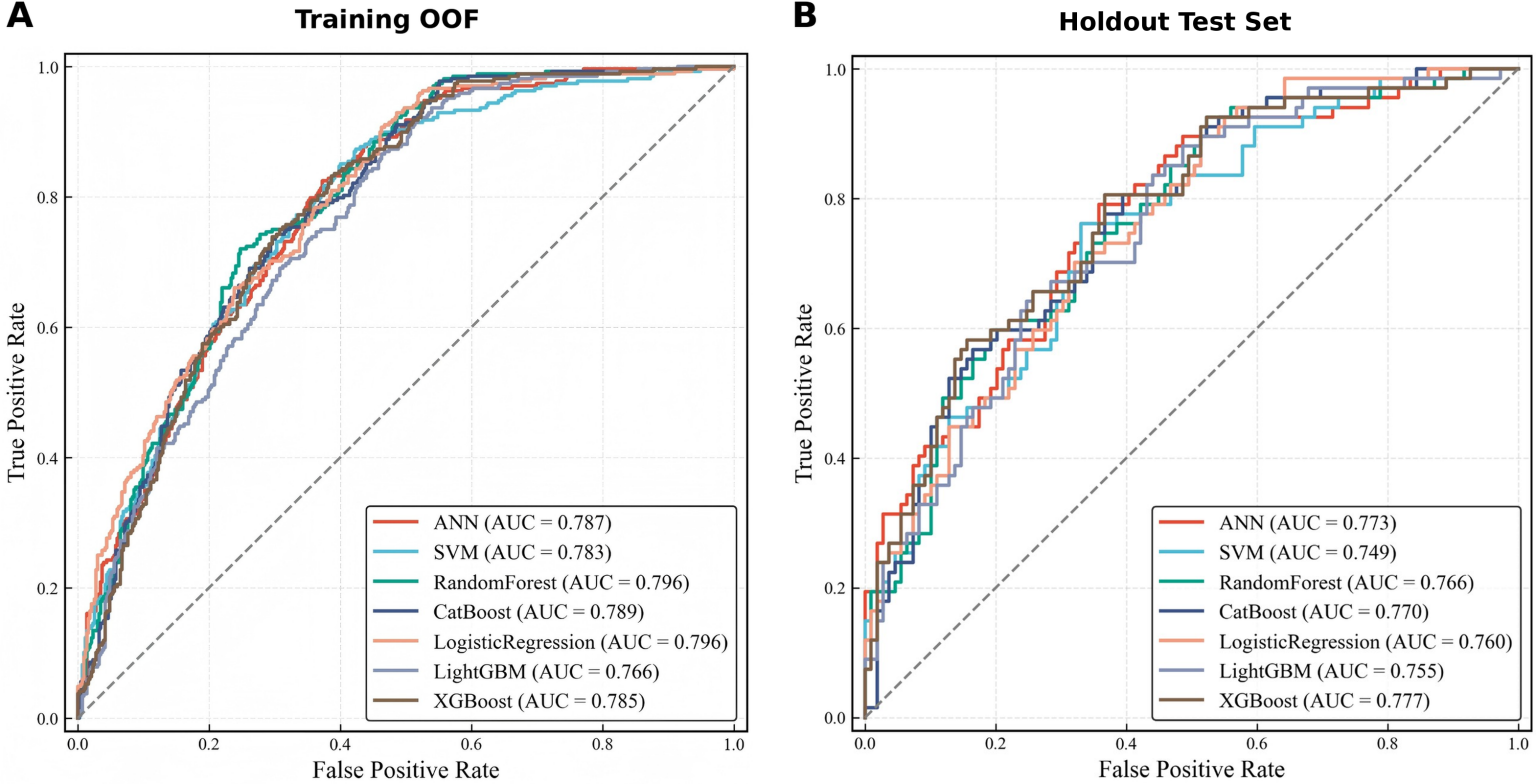
ROC comparison of seven machine-learning models. **(A)** ROC curves based on 5-fold cross-validation out-of-fold predictions in the training cohort. **(B)** ROC curves in the independent holdout test cohort. AUC values are shown in each panel. ROC, receiver operating characteristic; OOF, out-of-fold; AUC, area under the receiver operating characteristic curve; ANN, artificial neural network; SVM, support vector machine; CatBoost, categorical boosting; LightGBM, light gradient boosting machine; XGBoost, extreme gradient boosting.

As shown in **Figure 2A**, internal 5-fold cross-validation demonstrated that all models achieved moderate to good discrimination (AUC range: 0.766–0.796). LR and RF exhibited the highest internal discrimination (AUC = 0.796), followed closely by CatBoost (AUC = 0.789) and ANN (AUC = 0.787).

Upon evaluation on the independent holdout test set (**Figure 2B**), all algorithms retained meaningful discriminatory power beyond the training folds, with AUC values ranging from 0.749 to 0.777. While nonlinear models (XGBoost, AUC = 0.777; ANN, AUC = 0.773) showed marginally higher test AUCs than logistic regression (LR, AUC = 0.760), LR demonstrated comparable predictive performance with distinct advantages: full transparency, no black-box nature, and better compatibility with routine clinical reasoning. Accordingly, LR was selected as the final model for clinical translation.

### 4.4 Construction of the nomogram and feature importance

To facilitate intuitive clinical application, the full LR-derived nomogram was developed and is presented in **Supplementary Figure 1**. Global interpretability utilizing SHAP (SHapley Additive exPlanations) values and model coefficients revealed that the top predictors driving the risk of early acute postoperative HC included baseline biochemical indices (preoperative serum calcium and magnesium), patient characteristics (BMI), and specific surgical features.

**Figure 3:**
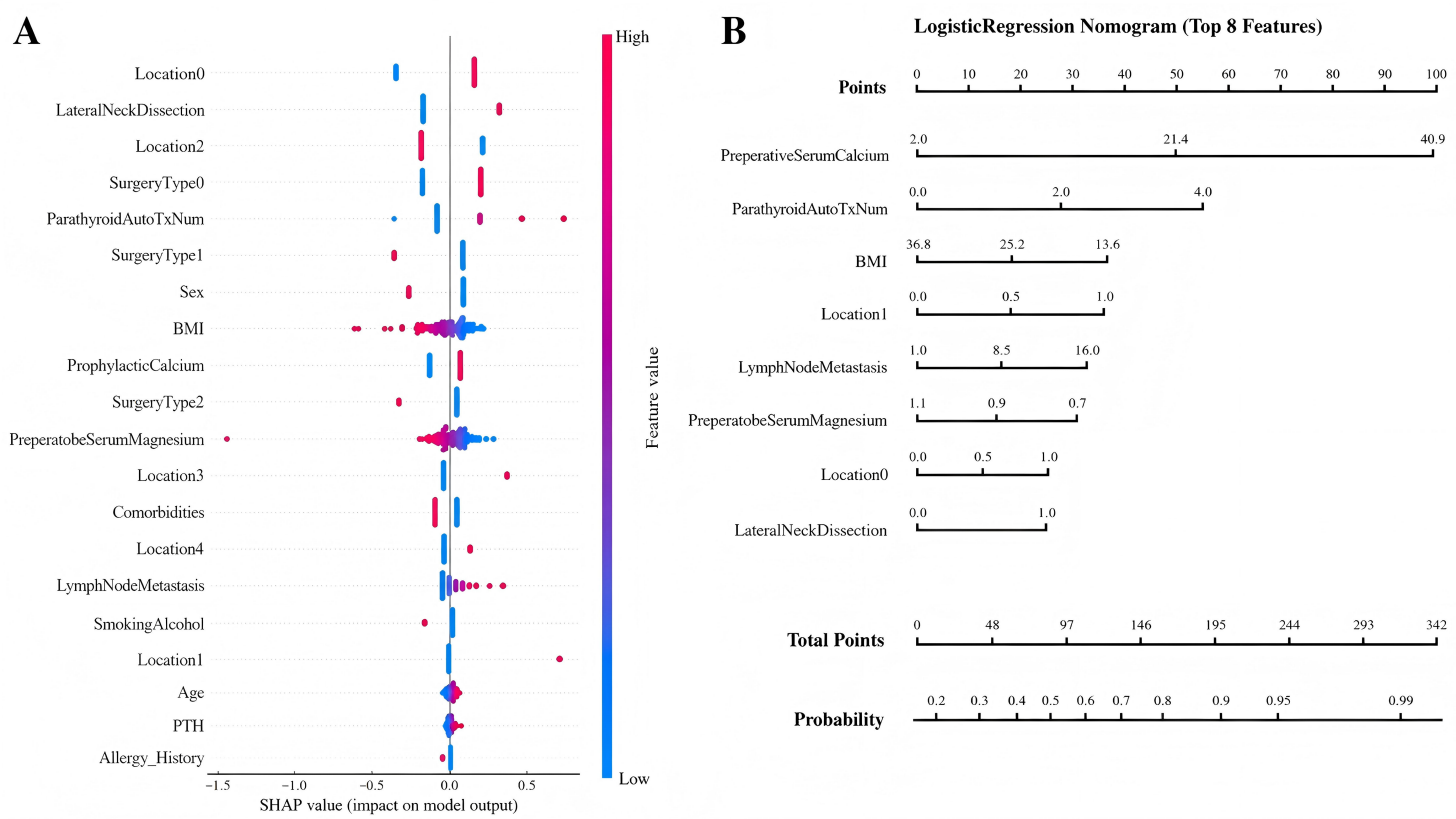
SHAP interpretation and simplified nomogram of the final logistic regression model. **(A)** SHAP summary plot showing the contribution of each predictor to the logistic regression model output. Positive SHAP values indicate increased predicted risk of early acute postoperative hypocalcemia, whereas negative values indicate decreased predicted risk. Red and blue dots indicate high and low feature values, respectively. **(B)** Simplified nomogram based on the top eight predictors for individualized risk estimation. SHAP, SHapley Additive exPlanations; BMI, body mass index; PTH, parathyroid hormone.

Global interpretability utilizing the SHAP summary plot confirmed that both preoperative patient characteristics and intraoperative surgical variables contributed meaningfully to the model’s predictive performance. Specifically, higher preoperative serum magnesium levels were associated with a reduced risk of hypocalcemia (negative SHAP values), while elevated BMI and age contributed to an increased risk (positive SHAP values). Furthermore, surgical factors—predominantly lateral neck dissection, specific surgery types, and parathyroid autotransplantation number—demonstrated consistent positive associations with the risk of hypocalcemia (**Figure 3A**).

Notably, confirming the findings of the multivariate analysis, parathyroid autotransplantation number and explicit implantation into the cervical muscle (Location 1) emerged as heavily weighted risk indicators on the nomogram. A standardized streamline nomogram incorporating these top 8 features was further presented to simplify bedside risk estimation (**Figure 3B**). Each predictor corresponds to a specific point on the scale, and the cumulative total points intuitively estimate the probability of developing early acute postoperative HC.

### 4.5 Threshold-based classification performance and calibration

**Figure 4:**
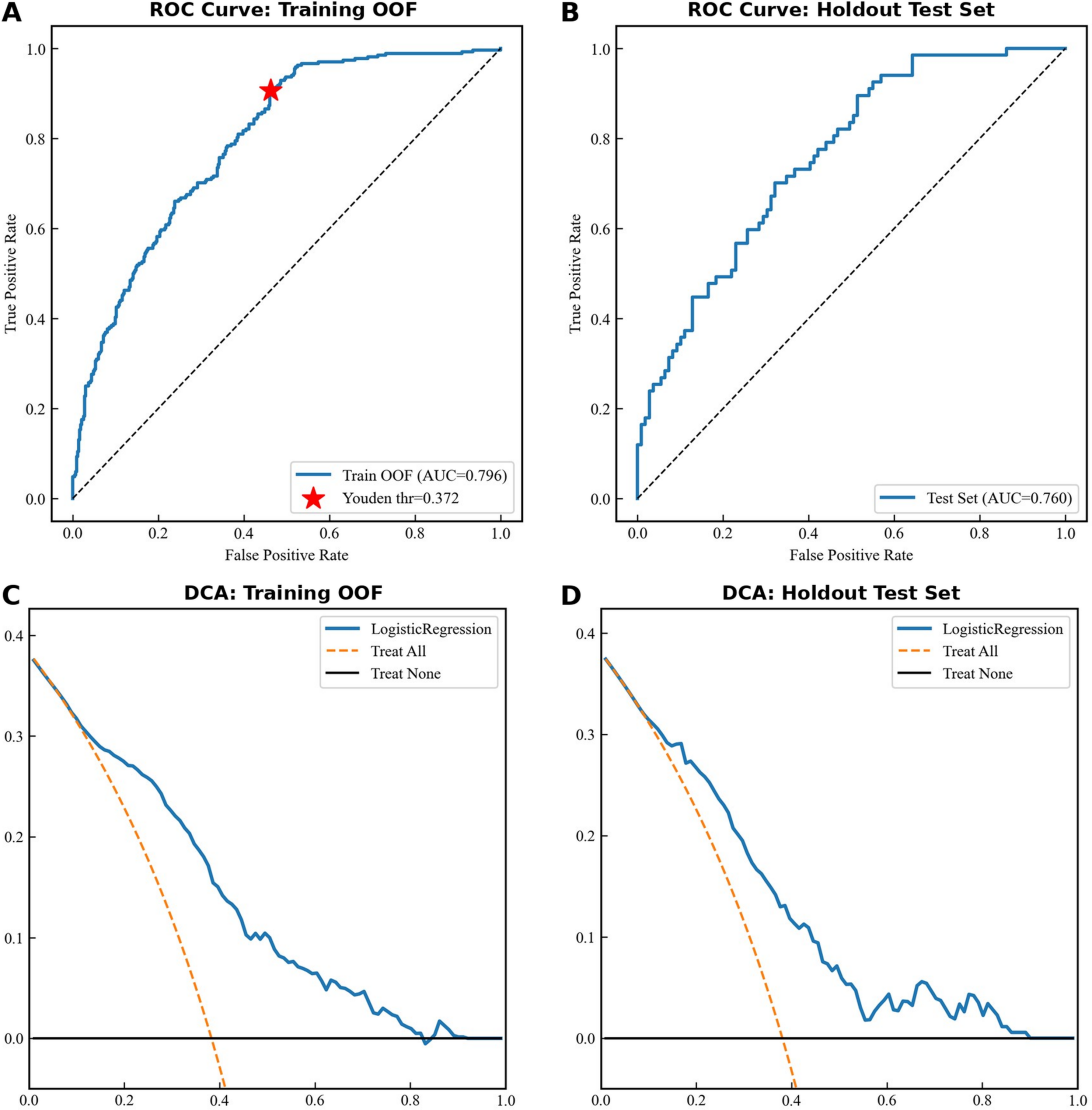
Discrimination and clinical utility of the final logistic regression model. **(A)** ROC curve in the training OOF cohort. **(B)** ROC curve in the independent holdout test cohort. **(C)** DCA in the training OOF cohort. **(D)** DCA in the independent holdout test cohort. ROC, receiver operating characteristic; OOF, out-of-fold; AUC, area under the receiver operating characteristic curve; DCA, decision curve analysis.

**Figures 4A, B** show the ROC curves for the logistic regression model in the training OOF cohort and independent holdout test cohort, respectively. The model achieved good discriminative performance, with an internal 5-fold cross-validation out-of-fold (OOF) area under the ROC curve (AUC) of 0.796 and an independent holdout test set AUC of 0.760, indicating limited overfitting and favorable generalizability. The Youden index identified an optimal threshold of 0.372 from the OOF cohort, yielding a high-sensitivity profile: sensitivity/specificity were 0.907/0.537 in the training set and 0.806/0.532 in the test set. At this fixed threshold, the accuracy, positive predictive value, and negative predictive value were 0.636, 0.514, and 0.817 in the independent holdout test set, respectively. This threshold effectively minimizes false negatives, which is critical for preventing severe symptomatic early acute postoperative hypocalcemia after thyroidectomy, while maintaining an acceptable false-positive rate.

Furthermore, DCA showed that the proposed LR predictive framework provides a greater net clinical benefit than both the alternative “treat-all” and “treat-none” strategies across a wide array of clinically relevant threshold probabilities in both the internal OOF cohort and the independent test set.This indicates that deploying the model can effectively increase the proportion of appropriately managed high-risk patients while limiting unnecessary interventions among low-risk individuals (**Figures 4C, D**).

### 4.6 Subgroup stability analysis

**Figure 5:**
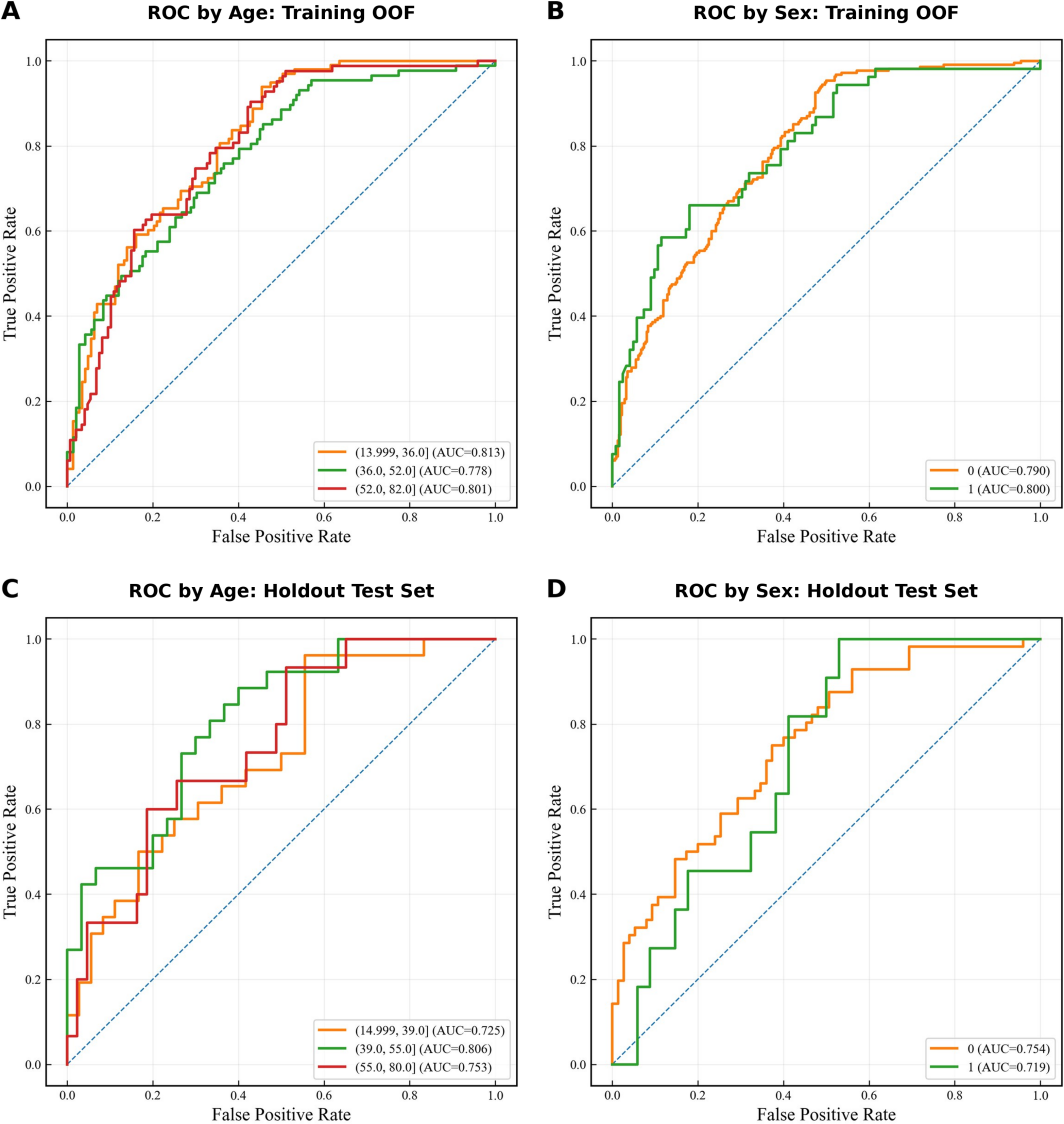
Subgroup analysis of model discrimination by age tertiles and sex. **(A)** ROC curves across age tertiles in the training OOF cohort. **(B)** ROC curves stratified by sex in the training OOF cohort. **(C)** ROC curves across age tertiles in the independent holdout test cohort. **(D)** ROC curves stratified by sex in the independent holdout test cohort. Age was stratified into tertiles for subgroup analysis. Sex was coded as 0 = female and 1 = male. ROC, receiver operating characteristic; OOF, out-of-fold; AUC, area under the receiver operating characteristic curve.

Subgroup analyses were performed to evaluate robustness across different demographic strata. In the training OOF evaluation, the model maintained discriminative power across age tertiles (AUCs: 0.813, 0.778, 0.801; **Figure 5A**) and sex (AUCs: 0.790, 0.800; **Figure 5B**).

On the holdout test set, the model’s discriminative capability remained robust and securely above chance level (Test AUCs for age: 0.725, 0.806, 0.753, **Figure 5C**; Test AUCs for sex: 0.754, 0.719, **Figure 5D**).

## 5 Discussion

Postoperative hypocalcemia poses a significant clinical and economic burden following thyroid surgery. The dilemma between indiscriminate prophylactic calcium administration and reactive “treat-only-when-symptomatic” strategies underscores the urgent need for precise risk stratification. In this study, abiding meticulously by the latest TRIPOD+AI guidelines, we evaluated seven machine learning algorithms on a robust cohort of 876 DTC patients. By bridging comprehensive clinical, biochemical, and specific surgical parameters, we developed and independently validated a highly discriminative Logistic Regression (LR)-based clinical nomogram for early acute postoperative HC within the first 24 hours after thyroidectomy (Holdout Test AUC = 0.760). Crucially, to our knowledge, this is one of the first studies to successfully quantify the physiological risk weight of specific parathyroid autotransplantation sites into a predictive nomogram framework.

To the best of our knowledge, this is the first study in the realm of post-thyroidectomy HC prediction to systematically evaluate and conduct a head-to-head comparison of seven distinct, mainstream machine learning algorithms. While the majority of existing literature relies on a single, pre-selected modeling approach (often exclusively conventional regression without algorithmic benchmarking (18)), our comprehensive pipeline ensured a highly rigorous methodological foundation. Through this extensive comparative analysis, we observed that while intricate ensemble methods—such as XGBoost and Artificial Neural Networks—demonstrated marginally superior discrimination in the initial evaluation, they intrinsically operate as mathematically opaque “black boxes.” (23) Therefore, rather than blindly pursuing the absolute highest AUC, we deliberately selected the LR model to construct the final nomogram. This decision guarantees maximal bedside interpretability and seamless integration into routine clinical workflows without sacrificing significant predictive power, perfectly aligning with modern calls for explainable AI in healthcare (24). Additionally, rigorous subgroup analyses substantiated that our model’s discriminative capability remained securely robust across varying age tertiles and sex strata in the test set. This confirms that the proposed nomogram is devoid of algorithmic bias and generalizes exceptionally well to diverse populations.

For clinical practice, this nomogram may serve as a point-of-care decision-support tool for thyroid surgeons and perioperative care teams. At the end of thyroidectomy or during the early postoperative assessment, clinicians can use readily available preoperative biochemical indicators, including serum calcium and magnesium levels, together with patient characteristics and intraoperative surgical information, such as lateral neck dissection and the number and site of parathyroid autotransplantation, to calculate a total point score and estimate the individualized risk of early acute postoperative hypocalcemia within the first 24 hours after surgery. Patients classified as high-risk may be prioritized for intensified serum calcium monitoring and proactive, targeted calcium supplementation to reduce the risk of symptomatic hypocalcemia. Conversely, for patients classified as low-risk, the nomogram may support streamlined postoperative monitoring and consideration of early discharge when combined with clinical symptoms, postoperative laboratory findings, and institutional discharge criteria. Therefore, this tool should be interpreted as an adjunct to, rather than a replacement for, clinical judgment in perioperative decision-making.

A paramount strength of our predictive model lies in accurately visualizing the relative impact of specific surgical dynamics. Consistent with clinical expectations, extensive neck explorations—such as lateral neck dissection—inevitably disrupt the delicate local microvascular network, contributing significantly to the risk profile. More innovatively, our nomogram explicitly demonstrates that the *number* of autotransplanted parathyroid glands acts as a dominant surgical predictor. The necessity of autotransplantation inherently implies glandular devascularization; thus, a higher number of grafts directly reflects a greater volume of transiently stunned parathyroid tissue. Furthermore, while autotransplantation into the cervical muscle (Location 1) also emerged as an independent risk contributor, its overall impact on the nomogram is relatively moderate. Biologically, the cervical musculature is situated directly within the surgical field, where local trauma and postoperative edema may transiently impair rapid angiogenesis (25). More importantly from a clinical perspective, heterotopic transplantation (e.g., into the forearm) is typically reserved for specific indications. According to clinical routines and guidelines, this explicitly prompts medical staff to initiate highly aggressive prophylactic calcium supplementation and rigorous PTH monitoring immediately post-surgery (26). This targeted clinical vigilance likely mitigates the manifestation of early postoperative hypocalcemia in these specific subgroups, effectively neutralizing their statistical risk in our cohort.

Consequently, extensive surgical disruption paired with multiple cervical autotransplantations should serve as a practical, heavily weighted clinical beacon for anticipating transient HC.

Beyond surgical mechanics, our nomogram further highlighted critical biochemical predictors. Preoperative serum calcium emerged as the most powerful independent predictor, carrying the greatest nomographic weight among all features. Patients with lower baseline calcium reserves possess a fundamentally diminished biochemical buffer, rendering them disproportionately vulnerable to any intraoperative parathyroid insult (27).In parallel, preoperative hypomagnesemia independently increases hypocalcemia risk. As magnesium is essential for PTH secretion and peripheral PTH responsiveness, preoperative hypomagnesemia severely impairs the compensatory PTH response to hypocalcemia (28).Collectively, these findings support a dual preoperative biochemical optimization strategy—correcting both serum calcium and magnesium—in high-risk surgical candidates.Additionally, higher BMI was associated with a significant protective effect against postoperative hypocalcemia, plausibly attributable to greater cervical adipose buffering against mechanical or thermal parathyroid injury, as well as greater adipose tissue storage capacity for fat-soluble vitamin D (27, 29).

Certain limitations warrant acknowledgment. First, as a single-center retrospective study, multi-center prospective validation remains necessary to confirm generalizability. Second, our cohort exclusively comprised conventional open thyroidectomy patients, reflecting our institutional practice; applicability to robotic-assisted and alternative-access approaches warrants future dedicated evaluation. Third, intraoperative PTH monitoring was not incorporated, as it does not constitute a routine component of our surgical protocol, reflecting real-world practice constraints. Finally, our endpoint was in-hospital early acute postoperative hypocalcemia within the first 24 hours after surgery. This model is not applicable to delayed, persistent, or chronic hypocalcemia beyond this period and long-term follow-up of persistent hypocalcemia beyond six months warrants prospective investigation.

## 6 Conclusion

In conclusion, we developed and validated a transparent, highly actionable nomogram for predicting early acute postoperative hypocalcemia within the first 24 hours after thyroidectomy in DTC patients. By uniquely integrating parathyroid autotransplantation profiles, preoperative serum calcium and magnesium, and key surgical parameters, this individualized tool enables accurate risk stratification. Clinical implementation, supported by DCA-confirmed net benefit, holds substantial promise for tailoring prophylactic calcium supplementation, enhancing patient safety, and streamlining postoperative management.

## Data Availability

The raw data supporting the conclusions of this article will be made available by the authors, without undue reservation.

## Supplementary material

Supplementary Figure 1 Full nomogram constructed from the logistic regression model for predicting early acute postoperative hypocalcemia. This logistic regression-based nomogram calculates the individualized risk of early acute postoperative hypocalcemia (within the first 24 hours) following thyroidectomy. Each variable corresponds to a specific score, and the total score is converted to the predicted probability of early acute postoperative hypocalcemia. BMI, body mass index; PTH, parathyroid hormone.

## References

[1] Han B Zheng R Zeng H Wang S Sun K Chen R . Cancer incidence and mortality in China, 2022. J Natl Cancer Center. (2024) 4:47–53. doi: 10.1016/j.jncc.2024.01.006 39036382 PMC11256708

[2] Cao B Wu G . Risk factors for hypocalcemia after total thyroidectomy: a narrative review. PeerJ. (2025) 13:e19808. doi: 10.7717/peerj.19808 40786097 PMC12333606

[3] Rocke DJ Mulder H Cyr D Kahmke R Lee WT Puscas L . The effect of lateral neck dissection on complication rate for total thyroidectomy. Am J Otolaryngol. (2020) 41:102421. doi: 10.1016/j.amjoto.2020.102421 32089352

[4] Silva BC . Skeletal and nonskeletal consequences of hypoparathyroidism. Arch Endocrinol Metab. (2022) 66:642–50. doi: 10.20945/2359-3997000000553 36382753 PMC10118831

[5] Bilezikian JP . Hypoparathyroidism. J Clin Endocrinol Metab. (2020) 105:1722–36. doi: 10.1210/clinem/dgaa113 32322899 PMC7176479

[6] Chen Y Forgetta V Richards JB Zhou S . Health effects of calcium: evidence from mendelian randomization studies. JBMR Plus. (2021) 5:e10542. doi: 10.1002/jbm4.10542 34761146 PMC8567492

[7] Rao SS Rao H Moinuddin Z Rozario AP Augustine T . Preservation of parathyroid glands during thyroid and neck surgery. Front Endocrinol. (2023) 14:1173950. doi: 10.3389/fendo.2023.1173950 37324265 PMC10266226

[8] Aras A Karayıl AR . Optimal surgical approaches for thyroid cancer: a comparative analysis of efficacy and complications. Med Sci Monit. (2024) 30:e942619. doi: 10.12659/msm.942619 38973140 PMC11302178

[9] Lalitha JJ Ramalingam N Rajan R Riju J Paulose AA Michael RC . Predictors of hypocalcaemia and hypoparathyroidism in patients undergoing thyroidectomy for benign and Malignant pathologies. Endocr Oncol. (2024) 4:e240022. doi: 10.1530/eo-24-0022 39649119 PMC11623251

[10] Moreno-Llorente P García-Barrasa A Pascua-Solé M Videla S Otero A Muñoz-de Nova JL . Usefulness of ICG angiography-guided thyroidectomy for preserving parathyroid function. World J Surg. (2023) 47:421–8. doi: 10.1007/s00268-022-06683-x 35945357

[11] Semanate F Tarupi W Fernandez Trokhimtchouk T Palacios C Jaramillo O . The role of parathyroid hormone level as a predictor of hypocalcemia after total thyroidectomy for thyroid cancer: a cross-sectional study. Cureus. (2025) 17:e78897. doi: 10.7759/cureus.78897 40091995 PMC11908629

[12] Soelling SJ Mahvi DA Liu JB Sheu NO Doherty G Nehs MA . Impact of obesity on risk of hypocalcemia after total thyroidectomy: targeted national surgical quality improvement program analysis of 16,277 patients. J Surg Res. (2023) 291:250–9. doi: 10.1016/j.jss.2023.06.006 37478649

[13] Collins GS Moons KGM Dhiman P Riley RD Beam AL Van Calster B . TRIPOD+AI statement: updated guidance for reporting clinical prediction models that use regression or machine learning methods. BMJ (Clin Res Ed). (2024) 385:e078378. doi: 10.1136/bmj-2023-078378 38626948 PMC11019967

[14] Yuan Y Zhang G Gu Y Hao S Huang C Xie H . Artificial intelligence-assisted machine learning models for predicting lung cancer survival. Asia-Pac J Oncol Nurs. (2025) 12:100680. doi: 10.1016/j.apjon.2025.100680 40201531 PMC11976224

[15] He Y Liu N Hao S Xu M Zeng Y . Interpretable machine learning-based prediction of mortality in critical cancer patients with delirium: a retrospective cohort study. Asia-Pac J Oncol Nurs. (2025) 12:100760. doi: 10.1016/j.apjon.2025.100760 40747249 PMC12311572

[16] Markus AF Kors JA Rijnbeek PR . The role of explainability in creating trustworthy artificial intelligence for health care: a comprehensive survey of the terminology, design choices, and evaluation strategies. J BioMed Inf. (2021) 113:103655. doi: 10.1016/j.jbi.2020.103655 33309898

[17] Zhou L Tu Y Qin S Zhang Q Ba H Wang P . Development and validation of a nomogram for predicting postoperative hypocalcemia in patients undergoing surgery for differentiated thyroid cancer. Front Endocrinol (Lausanne). (2025) 16:1628453. doi: 10.3389/fendo.2025.1628453 41122709 PMC12535898

[18] Cao B Zhang C Jiang M Yang Y Liu X . Development and validation of risk prediction models for permanent hypocalcemia after total thyroidectomy in patients with papillary thyroid carcinoma. Sci Rep. (2025) 15:9348. doi: 10.1038/s41598-025-93867-9 40102549 PMC11920412

[19] Wolff RF Moons KGM Riley RD Whiting PF Westwood M Collins GS . PROBAST: a tool to assess the risk of bias and applicability of prediction model studies. Ann Internal Med. (2019) 170:51–8. doi: 10.7326/m18-1376 30596875

[20] Muller O Bauvin P Bacoeur O Michailos T Bertoni M Demory C . Machine learning-based algorithm for the early prediction of postoperative hypocalcemia risk after thyroidectomy. Ann Surg. (2024) 280:835–41. doi: 10.1097/sla.0000000000006480 39109425 PMC11446540

[21] Antakia R Edafe O Uttley L Balasubramanian SP . Effectiveness of preventative and other surgical measures on hypocalcemia following bilateral thyroid surgery: a systematic review and meta-analysis. Thyroid Off J Am Thyroid Assoc. (2015) 25:95–106. doi: 10.1089/thy.2014.0101 25203484

[22] Orloff LA Wiseman SM Bernet VJ Fahey TJR Shaha AR Shindo ML . American thyroid association statement on postoperative hypoparathyroidism: diagnosis, prevention, and management in adults. Thyroid. (2018) 28:830–41. doi: 10.1089/thy.2017.0309 29848235

[23] Rudin C . Stop explaining black box machine learning models for high stakes decisions and use interpretable models instead. Nat Mach Intell. (2019) 1:206–15. doi: 10.1038/s42256-019-0048-x 35603010 PMC9122117

[24] Ali S Akhlaq F Imran AS Kastrati Z Daudpota SM Moosa M . The enlightening role of explainable artificial intelligence in medical & healthcare domains: a systematic literature review. Comput Biol Med. (2023) 166:107555. doi: 10.1016/j.compbiomed.2023.107555 37806061

[25] Cui Q Zhang D Kong D Tang J Liao X Yang Q . Co-transplantation with adipose-derived cells to improve parathyroid transplantation in a mice model. Stem Cell Res Ther. (2020) 11:200. doi: 10.1186/s13287-020-01733-4 32456711 PMC7249357

[26] Zhu JQ TWSA . Guidelines for perioperative parathyroid function protection in thyroid surgery (2018 edition). Chin J Pract Surg. (2018) 10:1108–13. doi: 10.19538/j.cjps.issn1005-2208.2018.10.03

[27] Mahvi DA Witt RG Lyu HG Gawande AA Nehs MA Doherty GM . Increased body mass index is associated with lower risk of hypocalcemia in total thyroidectomy patients. J Surg Res. (2022) 279:240–6. doi: 10.1016/j.jss.2022.06.002 35797751

[28] Karunakaran P Abraham DT Devadas G Hussain Z Kanakasabapathi R . The effect of hypomagnesemia on refractory hypocalcemia after total thyroidectomy: a single-center prospective cohort study. Indian J Endocrinol Metab. (2020) 24:518–24. doi: 10.4103/ijem.ijem_681_20 33643868 PMC7906105

[29] Fields T Ramonell K Fazendin J Gillis A Buczek E Porterfield J . The obesity paradox in thyroid surgery: is higher BMI protective against hypoparathyroidism? Am Surg. (2024) 90:9–14. doi: 10.1177/00031348231192065 37497666

