# Supplementary figures and images for: An interpretable nomogram for predicting early acute postoperative hypocalcemia in differentiated thyroid cancer: development and internal validation
Source: Front Endocrinol (Lausanne). 2026 Jun 17;17:1840443. doi: 10.3389/fendo.2026.1840443 (PMC13318600; doi:10.3389/fendo.2026.1840443)

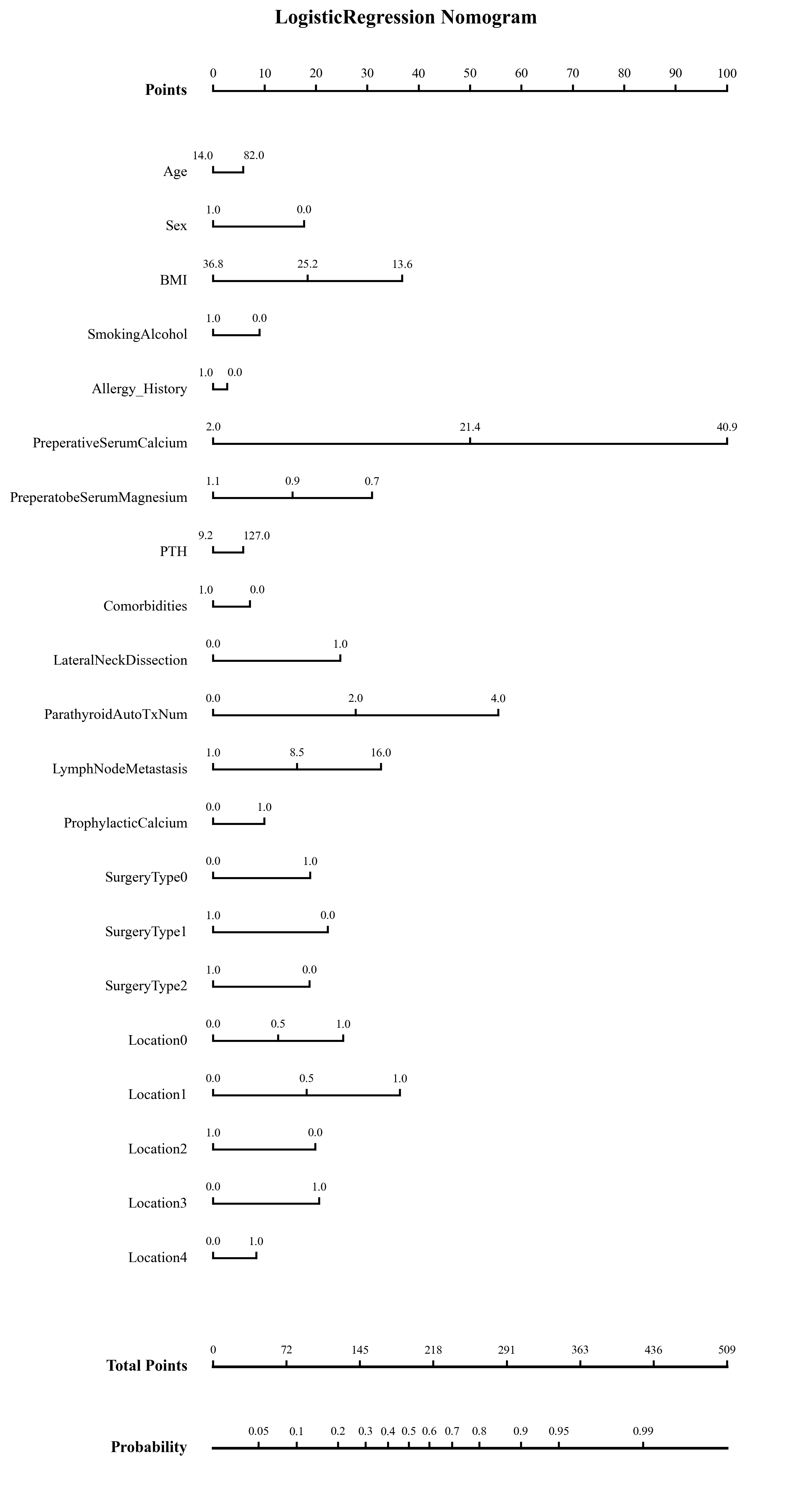

Supplement: Supplementary Figure 1 — Full nomogram constructed from the logistic regression model for predicting early acute postoperative hypocalcemia. This logistic regression-based nomogram calculates the individualized risk of early acute postoperative hypocalcemia (within the first 24 hours) following thyroidectomy. Each variable corresponds to a specific score, and the total score is converted to the predicted probability of early acute postoperative hypocalcemia. BMI, body mass index; PTH, parathyroid hormone. [file Image1.png]
